# Comparative evaluation between boric acid and sodium hypochlorite in pulp dissolving ability: An *in vitro* study

**DOI:** 10.6026/973206300214011

**Published:** 2025-11-15

**Authors:** Madhulika Singh, Anjali Gupta, Praveen Mishra, Abhishek Pal, Shruti Sharma, Shruti Kashyap

**Affiliations:** 1 Department of Conservative Dentistry and Endodontics, Maitri College of Dentistry and Research Centre, Anjora, Durg, Chhattisgarh, India-491001; 2 Department of Conservative Dentistry and Endodontics, Shri Balaji Institute of Dental Sciences, Raipur, Chhattisgarh, India

**Keywords:** Boric acid, sodium hypochlorite, pulp dissolution

## Abstract

Thorough elimination of pulp tissue remnants and significant reduction of microbial pathogens within the root canal system are essential for the success of endodontic treatment. Therefore, it is of interest to compare and assess the effectiveness of 10% boric acid, 5% boric acid and 5.25% NaOCl in dissolving pulp tissue. Forty pulp tissue samples obtained during routine endodontic procedures were standardized to 25 mg each, evenly distributed into four groups (n=10 per group). The initial weight of the samples was recorded using an analytical balance and each sample was then immersed in 5 ml of the respective solution for 30 minutes at room temperature. After immersion, the samples were re-weighed and the weight loss was recorded and analyzed.

## Background:

Effective root canal therapy hinges on meticulous chemo-mechanical preparation to eliminate microorganisms and pulp tissue. Irrigation solutions are essential during canal shaping, as they assist in debris removal, enhance antimicrobial action and promote tissue dissolution. Given the intricate anatomy of the root canal system, mechanical instrumentation alone is insufficient to achieve optimal cleaning and disinfection. Therefore, the use of appropriate irrigants is crucial to ensure the success of endodontic procedures [1]. Any remaining tissue within the root canal can serve as a reservoir for surviving bacteria, potentially leading to endodontic failure. Therefore, irrigation plays a vital role in thoroughly cleaning the canal [2]. Among various irrigants, sodium hypochlorite (NaOCl) is regarded as the gold standard due to its ability to dissolve pulp tissue remnants, eliminate the organic component of the smear layer formed during mechanical preparation and provide strong antimicrobial effects [3]. Grossman and Meiman stated that a 5% sodium hypochlorite (NaOCl) solution is capable of dissolving pulp tissue within a period ranging from 20 minutes to 2 hours. Dioguardi *et al.* emphasized that an effective irrigation protocol requires the use of a 5.25% sodium hypochlorite (NaOCl) solution for an adequate duration to achieve optimal results [4]. The anatomy of the root canal system is complex and varies in shape, making it difficult to remove organic tissue and reduce the microbial load, especially in the apical portion. This complexity can have a significant impact on the debridement process and the overall success of the RCT [5]. Boric acid is utilized as an irrigation agent due to its antiseptic, antibacterial and antifungal properties, along with notable anti-inflammatory and antimicrobial effects. While its impact on the smear layer and dentin mineral content has been studied, there is limited research on its ability to dissolve tissue [1]. A limited number of studies have been performed on the use of boric acid in dentistry. In one study that was conducted to treat periodontal disease, significantly less bone loss and less inflammation were reported with those who treated with boron [6]. Dakin's solution, a 0.5% sodium hypochlorite (NaOCl) formulation neutralized with boric acid, is one of the most commonly used irrigation solutions for primary teeth. It is preferred for its reduced irritation to peri-apical tissues compared to other irrigation methods [7]. 5.25% Sodium hypochlorite is quite effective in dissolving pulp tissue but it has many drawbacks like its irritant nature, unpleasant taste and odor, it is also harmful to periapical tissue. Prolonged exposure to NaOCl can compromise the mechanical properties of dentin, leading to reduced fracture strength and microhardness. On the contrary, Boric acid is odorless and tasteless, possesses mild antiseptic effects, which can aid in reducing bacterial load within the root canal system and has a less pronounced effect on dentin microhardness compared to NaOCl, potentially preserving the structural integrity of the tooth [8]. To check the effectiveness of boric acid in dissolving pulp tissue, this study was carried out. Therefore, it is of interest to compare and evaluate 5%, 10% Boric acid and 5.25% NaOCl for pulp tissue dissolution.

## Methodology: 

Forty pulp tissue samples were obtained from healthy premolars extracted for orthodontic and periodontal reasons. Each sample was standardized to a weight of 25 mg as shown in the Figure 1 (see PDF). The study utilized four distinct groups to evaluate the efficacy of various irrigation solutions on dental pulp tissue:

[1] Group A: 10% Boric Acid

[2] Group B: 5% Boric Acid

[3] Group C: 5.25% Sodium Hypochlorite

[4] Group D: Saline

The pulp tissue samples were initially rinsed with distilled water and gently dried using blotting paper. Their initial weights were recorded using a digital balance. Each sample was then immersed in 5 ml of the respective test solution for 30 minutes at room temperature. After immersion, the samples were dried again with blotting paper, re-weighed and the weight loss was calculated and analyzed.

## Statistical analysis:

The results were statistically analyzed by one way ANOVA and post hoc tuckey for pairwise comparison between the groups.

## Results:

Reduction in weight was highest with 5.25% Sodium hypochlorite followed by 10% Boric acid, 5% Boric acid & Normal saline as shown in the Figure 2 (see PDF). A Statistically significant difference in weight reduction was noticed between the groups. 10% BORIC ACID group displayed significant difference with 5% BORIC ACID and NORMAL SALINE group (p<0.05) but it gave a similar result with 5.25% NAOCL (p>0.05) of final weight.

## Discussion:

The capacity to dissolve pulp tissue is a fundamental attribute of an effective endodontic irrigant. Currently, sodium hypochlorite (NaOCl) solutions are the most widely used irrigants in endodontic therapy, primarily due to their unique tissue solvent effect and antibiofilm action [9]. Residual pulp tissue remnants can serve as a nutrient source for surviving bacteria, potentially leading to severe pain and compromising proper obturation, thereby contributing to endodontic treatment failure. Persistent or secondary intra-radicular infections are significant factors in such failures [10]. Boric acid has been recognized for its potential in dissolving organic tissue, flushing out infected material and neutralizing microbial byproducts. Its mild antiseptic properties make it effective in reducing bacterial load within the root canal system [7]. Boric acid (BA) has garnered attention as a potential endodontic irrigation agent due to its antifungal, antiseptic and potent antibacterial properties and proven biocompatibility with tissues. Studies have demonstrated that BA solutions, especially at concentrations around 10%, can effectively remove the smear layer and are advantageous in terms of ease of rinsing from the root surface [6]. Nisha *et al.* studied the efficacy of preprocedural boric acid mouth rinse in reducing viable bacteria in dental aerosols produced during ultrasonic scaling and concluded that routine use of preprocedural mouth rinses can help reduce bacterial aerosols generated during such procedures [11]. According to Hakki *et al.* Boron supplementation, whether in nutritional or supra-nutritional amounts, can be advantageous for the formation, maintenance and strength of bones and teeth in both animals and humans [12]. The present study used human pulp tissue samples to better simulate the oral environment, unlike similar studies that utilized bovine pulp. Additionally, the study employed smaller tissue samples due to the naturally lower weight of human pulp which is similar to the study done by Jain *et al.* [13].

In addition, weighing scale used in this study weighed the quantity of the pulp precisely. Denver measuring instrument, digital balance can quantify the pulp weight upto 4 decimals. Haapasalo *et al.* highlighted that an ideal irrigation solution should effectively dissolve both organic and inorganic tissue remnants. Given its potential properties, boric acid can be considered a suitable option for endodontic irrigation [14]. 10%, 5% Boric acid dissolves pulp but to a lesser extent than 5.25% NaOCl as shown in the Table 1 (see PDF) and Table 2 (see PDF). Similar findings were established in the study done by Cakir *et al.* where 5% Boric acid ranked behind NaOCl [1]. This is the first study to use 10% boric acid for pulp dissolution. Sodium hypochlorite exhibits a saponification reaction, chloramination reaction and neutralization reaction. When chlorine dissolves in water and comes into contact with organic matter, it generates hypochlorous acid, which functions as a powerful oxidizing agent [15]. According to Nisha *et al.* Boric acid possesses antifungal and antibacterial properties, making it a candidate for inclusion in mouthwashes [11]. It also possesses anti-microbial and anti-inflammatory properties. Boric acid has demonstrated the ability to suppress bacterial growth, including that of *Enterococcus faecalis*, a prevalent pathogen in endodontic infections. Additionally, it possesses antifungal properties, making it effective against *Candida albicans*, a fungus associated with endodontic infections hence the various factors including antibacterial, antifungal and antibiofilm properties, toxicity, tissue solubility, substantivity, effects on dentin and the smear layer, as well as potential side effects like allergic reactions play a role in selecting a suitable irrigant [16, 17]. Boric acid dissolves pulp tissue in endodontics especially through its acidic property which leads to protein denaturation and tissue breakdown. It can also draw water out of the pulp tissue through osmosis, causing the pulp to shrink and thus making it easier to remove [1]. Boric acid has anti-inflammatory properties, which can help reduce inflammation [18]. Alici *et al.* have found that a 6% boric acid solution serves as an efficient root canal disinfectant, exhibiting notable antibacterial activity against *E. faecalis* biofilm. While its effectiveness is comparable to that of sodium hypochlorite, the highest concentration of boric acid (6%) showed the strongest bactericidal properties, though it did not achieve the same level of effectiveness as NaOCl. Nevertheless, boric acid demonstrates significant bactericidal potential [19]. Güçlüer *et al.* found Sodium hypochlorite to demonstrate the highest tissue-dissolving capability among all tested solutions. Additionally, butanol and methanol extracts of *Sapindus mukorossi* were also found to be effective in dissolving pulp tissue [20].

## Conclusion:

5.25% NaOCl is effective as pulp dissolving agent than 10 and 5% Boric acid. In vital cases, boric acid should not be used as a pulp dissolving agent but can be used as an endodontic irrigant. Further research is needed on boric acid to evaluate its pulp dissolution efficacy.
